# Why Exercise at Work: Development of the Office Exercise Behavior Determinants Scale

**DOI:** 10.3390/ijerph18052736

**Published:** 2021-03-08

**Authors:** Tianmei Zhang, Jaap Ham, Xipei Ren

**Affiliations:** 1 School of Design and Arts, Beijing Institute of Technology, Beijing 100081, China; t.tianmei.zhang@gmail.com; 2 Department of Industrial Engineering and Innovation Sciences, Eindhoven University of Technology, 5600 MB Eindhoven, The Netherlands; j.r.c.ham@tue.nl

**Keywords:** office exercise, behavior determinants, workplace health promotion, questionnaire

## Abstract

The constant increase in work pressure and the penetration of labor-saving technologies have significantly reduced physical activity in office-based work routines, threatening employees’ physical and mental well-being. Encouraging physical exercises at the office seems a potential solution. However, while there is a wealth of research into occupational health and workplace exercise promotion, little is known about which factors can influence the engagement of physical exercises in the office context. It is crucial to understand these determinants, in order to support the design of office exercise promoting intervention. This study explored the determinants of office workers’ exercise behavior by proposing and developing the Office Exercise Behavior Determinants (OEBD) scale based on existing behavioral and environmental research. The OEBD scale was assessed through an online questionnaire study involving 479 office workers. The results indicated that four factors (Intrinsic Motivation, Extrinsic Motivation, Social Environment, and Work Environment) contribute to office workers’ exercise behavior. Furthermore, confirmatory factor analysis of our obtained data provided evidence for the internal validity of the OEBD scale. Thereby, this research can support increased office exercise with valid measurements for behavioral determinants.

## 1. Introduction

Since the Industrial Revolution in the 19th century, the invention of labor-saving equipment has led to a continuous decrease in physical exercise [1,2]. During recent decades, the penetration of information communication technologies and the popularization of internet-based services has diminished the necessity of physical movement (e.g., business travel, or a walk to a colleague’s office) and made it possible for people to complete most of their work while seated [3]. This operating mode improved the efficiency of work, but also substantially reduced physical exercise and increased sedentary behaviors [1,4]. According to the WHO, 60% to 85% of people in the world live in sedentary lifestyles without sufficient physical exercise [5], making lack of physical activity one of the most serious health problems across different ages [6]. Beyond that, task-oriented working norms and high-pressured working environments have exacerbated office workers’ inactivity at work [7].

Recent studies have shown an increase in sedentary behaviors in the working environment [8,9]. In most office environments, it is tacitly approved that office-based employees stay in sedentary conditions at most times during the working day [10,11,12]. The International Labor Organization reported that working hours are generally regulated to 8 h per working day [13], and are mostly spent in sedentary conditions [3,14]. Earlier research suggested that prolonged physical inactivity at work can dramatically increase the risks of developing many occupational diseases and injuries [15]. For instance, low levels of physical activity are increasingly recognized as important contributors to a variety of health problems, including heart disease, hypertension, colorectal cancer, obesity, and osteoporosis [16,17,18]. Sedentary lifestyles are also significantly associated with an increased incidence of psychosocial problems, including depression, stress, and loneliness [19,20]. Additionally, sedentary behaviors can lead to low back pain, neck discomforts, chronic shoulder problems, and many other musculoskeletal injuries [21,22].

When focusing on determinants of physical exercise at the workplace, earlier research has shown that workplace physical exercises can improve the health conditions of office-based workers who sit a lot [23,24]. Many previous studies have been focused on investigating exercise interventions for office workers using e.g., public policies [25], health programs [26], environmental change [27], social supports [28], motivational materials [29], digital tools [30], expert consultations [31], etc. Yet little is known about which factors influence participation in workplace exercise behavior [15,32,33]. There are many barriers to physical exercise at the office, such as lack of time to perform physical activities [34], work burden and performance concerns [35], workplace policies and norms [36]. For instance, performing physical exercises in the workplace may be heavily influenced by colleagues’ and superiors’ behavior and attitude [34]. There are also several limitations in the office environment, such as lack of public space and public facilities [36,37,38]. The barriers indicated above are crucial to understanding office workers’ exercise behavior. However, to our knowledge, there is a lack of research on identifying those barriers and their influences on the participation in exercise at the office.

In this paper, we investigated the determinants of office exercise behaviors for supporting workplace fitness interventions. Based on the review of related work, we developed a scale for measuring office exercise behavior determinants, which contained 18 potential determinants related to individual and socioenvironmental characteristics to determine the involvement of physical exercise at the office. We conducted a study in which 479 participants were asked to fill out a questionnaire consisting of items for assessing these potential determinants in order to develop the list of items for the instrument and to initially validate this scale.

## 2. Materials and Methods

### 2.1. Materials

#### 2.1.1. The Office Exercise Behavior Determinants

Following Sallis & Hovell [3], Owen et al. [32], and Giles-Corti & Donovan [39], in this project we took an ecological view to investigate individual and environmental characteristics determining physical exercise behaviors in the office. Regarding the determinants of individual characteristics, we drew on the renowned cognitive frameworks and related studies closely connected to exercise motivation, including the Theory of Planned Behaviour [40], the Self-Determination Theory [41], Self-Efficacy [42], Transtheoretical Model [43], and the Health Belief Model [44]. Regarding the determinants of environmental characteristics, as suggested by Owen et al. [3], the social ecological perspective is needed to understand the context specific determinants for intervening in inactive behaviors. Similarly, this study also explored both the social (e.g., [45,46]) and physical environments (e.g., [47,48]) regarding office exercise behaviors. After several iterations of internal discussion between co-authors, as well as consultations with domain experts, we synthesized 18 potential determinants of office exercise behaviour based on the constructs of those aforementioned theories and relevant studies [40,42,49,50,51,52,53,54,55,56,57,58,59,60,61].

As shown in Table 1, we listed these 18 potential determinants in the first column, with their references in the second column and the existing corresponding measures in the third column.

To measure the 18 potential determinants, we developed 52 items in two ways. First, for determinants which had existing measures, we adapted the items according to the office exercise context (i.e., Perceived Behavioral Control, Appearance, Family and Friend Influence, Social Support, Social Reinforcement). Second, for determinants that had no existing measures, we created items based on measures used in a similar context (i.e., Colleague Influence, Superior Influence, Public Space Scale, Exercise Facilities, Exercise Tutorial, Vitality Tradition, Work Pace, Break Time, Policy of Working Company, Work Burden). For instance, to measure the determinant Public Space, we developed the item *“For me, having enough public activity space for exercise in the office is important”* based on the study by Taylor and Todd [53]. 

In this project, all the items were measured by a 7-point Likert Scale (from 1 = strongly disagree to 7 = strongly agree). Specifically, Perceived Behavioral Control included 3 items adapted from the items developed by Ajzen & Madden [62]. Competence included 7 items employed originally from Buckworth et al. [63]. Perceived Health included 9 items employed originally from Sechrist et al. [64]. Enjoyment was measured using 8 items employed originally from Buckworth et al. [63]. Appearance contained 5 items adapted from Buckworth et al. [63]. Family and Friends’ Influence was measured using 2 items adapted from Taylor & Todd [54]. Colleague Influence (2 items) and Superior Influence (3 items) were created referring to items used in Family and Friends’ Influence. Social Support included 2 items adapted from Buckworth et al. [63] and Sechrist et al. [64]. Social Reinforcement was measured using 1 item adapted from Elliot et al. [65]. Public Space Scale (2 items), Exercise Facilities (2 items), and Exercise Tutorial (1 item) were created referencing the items Taylor & Todd [53] developed. Vitality Tradition was measured using the item “The healthy tradition at my office influences my exercise behavior.” The same type of created items were used in measure Work Pace, Break time, Policy of Working Company, and Work Burden.

#### 2.1.2. The Questionnaire for Assessing Office Exercise Behavior Determinants 

To investigate the internal validity of this scale, we developed a questionnaire for an online survey study. The questionnaire was designed in two parts. The first part contains the scale with all the items for measuring office exercise behavior determinants. Additionally, we also wanted to explore the relationship between these behavior determinants and people’s intentions to perform office exercise, which is crucial to predicting health behavior [66]. In this study, we adopted three items from McAuley and Jacobson [67] to measure participants’ behavior intention. They are: “I intend to do physical exercise. (1 = extremely likely, 7 = extremely unlikely)”; “I will try to do physical exercise in my office. (1 = extremely likely, 7 = extremely unlikely)” and “How regularly do you intend to do physical exercise in your office? (measured by 4-point Likert Scale, always-rarely)”. 

The second part of the questionnaire comprised various questions to assess participants’ physical activity, occupation, and demographic information. Specifically, physical activity conditions were assessed with the Godin-Shephard Leisure-Time Physical Activity Questionnaire [68,69], which has been widely validated and applied in physical activity research [70,71]. Here, we asked the participants to indicate weekly frequency of strenuous, moderate, and light exercises respectively, which were then used to calculate a weekly leisure activity score based on the following formula:

Score = (9 × Strenuous) + (5 × Moderate) + (3 × Light)

The score could be used to identify the participant’s health level as insufficiently active (0 to 14), moderately active (14 to 23), or active (24 or more) [69]. In addition, the participant’s experience of exercise in the workplace was measured with the question: “Have you ever performed physical activities during work time?” and the working environment was measured with the question: “What is your working environment?”. We also asked respondents to provide occupation information including occupation conditions, occupation role, working industry, working organization, and working hours per week. Participants’ gender, age, and length of education were also collected through several single option questions adopted from Hofstede et al. [72]. All details of the questionnaire used for the online survey can be found in Appendix A.

### 2.2. Recruitment

The questionnaire was distributed in two online databases, Prolific and WenJuanXing, to recruit respondents who had an office-based job for more than 30 h per week. Prolific (https://www.prolific.co/; accessed on 7 January 2020) is an online platform that collects high-quality responses from people around the world while WenJuanXing (https://www.wjx.cn/; accessed on 26 January 2020) is an online research platform widely used in China. The study was conducted according to the guidelines of the Declaration of Helsinki and approved by the Institutional Ethics Committee of Eindhoven University of Technology (protocol code 1016 and approval on 11 December 2019). The participation of this study was fully voluntary. Upon the completion of the questionnaire, the participant was thanked with a value of €1.40 payback from the platform.

### 2.3. Procedure

Participants were invited to the study through the Prolific and the WenJuanXing database systems. The platforms sent an e-mail to participants with a link to the questionnaire and detailed instructions. This link allowed participants to fill out the questionnaire on a variety of devices (e.g., computer, smartphone, etc.). Before filling in the questionnaire, the participant was presented with a welcome message containing the introduction, the estimation of completion time, and the study instructions. The participant was asked whether they would agree to the terms of the consent form. Upon agreement to take part in this study, the participant was invited to complete the questionnaire. At the end of the questionnaire, the participants were reminded again about their rights and privacy. 

### 2.4. Data Analysis

Questionnaire responses were analyzed using IBM SPSS version 22.0 (IBM, Armonk, NY, USA). Similar to Buckworth et al. [63] and Gerbing et al. [73], we initiated the quantitative analysis with an internal reliability test of those initial determinants. We applied principal component analysis with varimax rotation based on the following procedure [74,75]. Firstly, we eliminated items that had cross-loading or had weak correlation with other items in a common factor [76]. Secondly, we iteratively eliminated items that had a factor loading value less than 0.5 [77,78]. Thirdly, after Bartlett’s test of sphericity [79] to verify the sampling adequacy of the remaining items, we conducted the final exploratory factor analysis for internal reliability and for finalizing the items of the scale.

Next, we explored the underlying factors of the developed instrument based on principal component analysis [73] and Varimax with Kaiser Normalization rotation method [75]. The identified factor structure was further evaluated through a confirmatory factor analysis [80] using IBM AMOS 22.0 (IBM, Chicago, IL, USA). Specifically, the fit of the confirmatory factor models was assessed using the indicators comparative fit index (CFI), the root mean square error of approximation (RMSEA), and the standardized root mean square residual (SRMR) [81,82]. The fit of the models was statistically compared by testing the differences in χ^2^. Furthermore, the convergent validity of the identified model was evaluated using the average variance extracted (AVE) [83] and composite reliability (CR) [84], while its discriminant validity, was assessed based on the square root values of the AVE. We eventually performed several quantitative analyses, such as Chi-Square and regression tests to exploratively analyze the relationships between different measures of our questionnaire.

## 3. Results

### 3.1. Participants’ Description

A total of 512 responses were received from Prolific (N = 209) and WenJuanXing (N = 303) and 33 responses were excluded due to the following reasons: (a) occupation condition was selected as “retired”; or (b) working environment was selected as “outdoor” or “home”; or (c) working hours were less than 30 h per week; or (d) gave two opposite answers on the same statement (appearing on a different part of the questionnaire). Therefore, the questionnaire responses from 479 participants (238 female, 240 male, and 1 non-binary) were considered as valid data and used for analyses. Most of the participants (72.7%) had finished 16 or more years of education. The majority of participants (69.9%) were physically active indicated by a score of more than 24 on the Godin-Shephard Leisure-Time Physical Activity Questionnaire [68,69]. The rest of the sample was moderately active (13.4%) or insufficiently active (16.7%). Most of the participants (77.2%) had performed physical activities in the workplace at least once, whereas 109 participants (22.8%) had never performed physical activities in the workplace. Most of the participants (88.1%) had paid fulltime work, and 84.5% of the participants worked in an office during all working hours. Of these participants, 76.8% worked more than 40 h per week. Most of the participants (68.5%) were working in the private sector, while 21.9% of the participants were working in the public sector, 5.2% of the participants for the non-profit sector and the rest (4.4%) chose their organization type as “others”. Of all the participants, their working industry ranged from manufacturing (17.3%) to professional, scientific and technical services (15.9%), education (11.7%), information (9.4%), finance and insurance (8.8%), government and public administration (5.6%), and so forth. Overall, the sample was representative of office workers who have relatively long working hours in an office-based environment, across gender, working industry, occupation role, prior exercise experience, and physical activity level. 

### 3.2. The Office Exercise Behavior Determinants Scale Development

Following the procedure described in the previous section, we investigated the internal reliability of those initial determinants using exploratory factor analyses. We eliminated 9 items (9, 10, 11, 31, 38, 39, 48, 49, 50) that had cross-loading or had weak correlation with other items in a common factor and 11 items (26, 36, 37, 40, 41, 51, 52, 53, 54, 55, 56) that had a loading value less than 0.5. After eliminating those items, we had 32 items left. In our Bartlett’s test, the Kaiser-Meyer-Olkin measure of sampling adequacy was 0.90, which indicated the 32 items were adequate for exploratory factor analysis, as suggested by Cattell [85] and Kaiser [86]. 

The final exploratory factor analysis showed that all the 32 items meet the following criteria: (a) loading value > 0.5 [78], (b) no cross-loading, (c) communality value > 0.5 [87], (d) the cumulative contribution rate of the factor model as large as possible [88] and (e) the extracted factor model was both transparent and interpretable from a professional perspective [89]. That suggests that the intercorrelation of the 32 items was reliable and these items could well represent the corresponding determinant. 

With the analyses described above, we developed the Office Exercise Behavior Determinants (OEBD) scale, which retained 32 items representing 10 determinants. That is, 8 of the initial 18 determinants were removed as they had lost all their measurement items [85,90]. Thereby, 10 determinants remained that had items which satisfied the criteria of round four described above. All determinants and items of the newly developed OEBD scale can be found in Appendix B (Table A1).

### 3.3. The Factor Structure Investigation

Through exploring the dimensionality of the retained 32 items of the OEBD scale, a clear four-factor solution was obtained with three criteria: (a) eigenvalues greater than 1.00 [91], (b) examination of the screen plot [92], and (c) factor solution interpretive and theoretically sensible [80]. As shown in Table 2, the four-factor solution accounted for 60.46% of the total variance. Table 3 shows that the factor loadings on each factor were between 0.66~0.84 and with no cross-loading, indicating that the factor structure is clear [78]. Hence, this four-factor solution was considered appropriate with the substantive interpretability. The Cronbach’s alpha for each factor was greater than 0.84, thus indicating sufficient reliability [93].

As shown in Table 3, the four-factor solution indicated four interpretable factors. Factor 1 included thirteen items that reflected the determinant of Competence and Enjoyment, which we termed as Intrinsic Motivation. Factor 2 included eight items that reflected the determinant of Perceived Health, which we termed as Extrinsic Motivation. Factor 3 included six items that reflected the determinants of Colleague Influence, Superior Influence, and Family and Friend Influence, which we termed as Social Environment. Factor 4 included four items that reflected the determinants of Work Pace, Work Burden, Break Time in Office, and Policy of Working Company, which we termed as Work Environment. 

### 3.4. The Scale Structure Assessment

We performed a confirmatory factor analysis to explore the factor structure of the OEBD scale. To do so, we constructed a four-factor model (the target model, based on the four factors coming out of the exploratory factor analyses described above) and three competing models. We performed confirmatory factor analysis to test whether we can statistically distinguish the following four models.

- Model A: a one-factor model with all items assumed to load on one factor;
- Model B: a two-factor model with separate factors for motivation (Intrinsic Motivation + Extrinsic Motivation) and environment (Social Environment + Work Environment);
- Model C: a three-factor model with separate factors for motivation (Intrinsic Motivation + Extrinsic Motivation), Social Environment, and Work Environment;
- Model D: a four-factor model with separate factors for Intrinsic Motivation, Extrinsic Motivation, Social Environment, and Work Environment.

As can be seen in Table 4, the results of confirmatory factor analysis showed that Model D with four latent factors had a significantly better fit than the other three models with an acceptable overall fit (CFI = 0.817, RMSEA = 0.089, SRMR = 0.073). This implies that the four-factor structure of the OEBD scale fitted the data better than a three-factor, a two-factor, or a one-factor structure.

Regarding the convergent validity of the four-factor model, Table 5 shows that the composite reliability of all constructs was greater than 0.7. Among the four factors, three of the AVE values were greater than 0.5. These results indicated an acceptable convergent validity, as suggested by Hair et al. [77] and Chin [94].

Regarding the discriminant validity of the four-factor model, Table 6 reported that the square root values of AVE were 0.75, 0.65, 0.74, and 0.76. The minimum value of the AVE square root was 0.65, which is greater than the maximum value of all correlation coefficients (0.37). The results indicated that the four-factor structure had satisfactory discriminant validity [83].

Overall, the four-factor model of the OEBD scale had a satisfactory convergent validity, discriminant validity, and a good overall fit. The four-factor structure found in the exploratory factor analysis was assessed with the confirmatory model.

### 3.5. Exploratory Analyses

We performed a Chi-Square test to examine the relationship between physical activity (measured by the Godin-Shephard Leisure-Time Physical Activity Questionnaire) and working conditions (measured by item 2–8). The result showed that there was no significant relationship between participants’ physical activity and their work type, work industry, work role, work organization type, work environment, and work hours per week. Only one variable “experience in physical activities during work time” showed a relationship with physical activity (χ^2^ = 16.11, *p* < 0.001), which means that participants who had performed exercises during work time had a higher level of physical activity.

We performed a regression analysis to investigate the relationship between the four common factors and exercise behavior intentions. During the analysis, we took each item for the exercise behavior intentions as the dependent variables and the four newly identified factors as independent variables. The results showed that Intrinsic Motivation (Beta = 0.40), Extrinsic Motivation (Beta = 0.28), and Work Environment (Beta = 0.17) had significantly positive effects on office worker’s exercise behavior intention in general (measured by item 61), due to an adjusted R^2^ of 0.34 (*F* = 63.43, *p* < 0.001). Additionally, Intrinsic Motivation, Social Environment, and Work Environment had significantly positive effects on participants’ exercise behavior intention in the office context, as the regression resulted in the adjusted R^2^ of 0.20 (*F* = 29.90, *p* < 0.001) (item 62 as the dependent variable) and 0.15 (*F* = 22.28, *p* < 0.001) (item 63 as the dependent variable), respectively.

## 4. Discussion

Promoting exercise behavior in the workplace is a potential solution for physical inactivity problems among office workers. Understanding which determinants impact on office workers’ exercise behavior is crucial for office exercise promoting intervention. We argue that general exercise determinants cannot comprehensively explain office workers’ exercise behavior in the specific office context. Therefore, this study investigated the determinants of office exercise behavior. By performing a literature study, we identified 18 potential determinants with corresponding 52 items to measure. To assess these determinants, we conducted an online survey and collected valid questionnaire response data from 479 office workers globally. Then we performed an exploratory factor analysis to further develop and validate the Office Exercise Behavior Determinants (OEBD) scale. At the same time, we used this exploratory factor analysis to find common factors underlying these determinants. After an appropriate factor structure was found, we performed a confirmatory factor analysis to assess the structure of the OEBD scale. 

This OEBD scale consists of 32 items representing 10 determinants. We were also able to identify four underlying factors of the OEBD scale. That is, an exploratory factor analysis of OEBD scale suggested a four-factor solution, consisting of Intrinsic Motivation, Extrinsic Motivation, Social Environment, and Work Environment. With most items loading strongly onto each factor, results showed that the four-factor structure accounted for more than 60% of the total variance. Specifically, the Intrinsic Motivation items reflected determinants Competence and Enjoyment. The Extrinsic Motivation reflected determinant Perceived Health. The Social Environment encompassed items that involved determinants Colleague Influence, Superior Influence, and Family and Friends’ Influence. The Work Environment items reflected determinants Work Pace, Work Burden, Break Time, and Policy of Working Company. These items were substantively congruent with their respective factors, and the strong factor loadings provided further evidence for the viability of these factors. 

The current results confirmed and extended earlier studies on exercise determinants. Dishman et al. [54] suggested that strong determinants of exercise participation are self-motivation, behavioral skills, perception of good health, and available time. In the current study, we also confirmed that enjoyment, competence, perceived health, and break time are exercise determinants in the worksite. Earlier findings also showed that support from people in home and work environments is another robust correlate of exercise behavior [44,45,95]. In the current study, we confirm that family and friends’ influence, colleague influence, and superior influence are determinants for office workers’ exercise behavior. Besides that, three determinants (work pace, policy of working company, and work burden) extended the exercise determinants study in a worksite specific manner.

The current results found four underlying factors, which were also found in earlier research. That is, earlier studies likewise provided evidence that Intrinsic Motivation, Extrinsic Motivation, and Social Environment are essential to exercise behavior [96,97,98]. Besides, the current results extended earlier findings. That is, current results showed that Work Environment as included in the newly developed OEBD scale correlates with office-based exercise behavior. This implies that specific determinants in the office context (such as work burden, work pace, and break time in office) are associated with exercise behavior in the office. The factor Work Environment needed to be considered in office exercise behavior promoting programs as an important factor. 

The assessment of a confirmatory model confirms the four-factor structure found in the OEBD scale. We compared four models based on the model fit indices. Examination of χ^2^ suggested that the four-factor model adequately described the scale better than the other three models. The proposed four-factor structure lends supportive reliability and validity evidence to the OEBD scale.

Furthermore, exploratory analyses suggested that when a participant performed physical activities during work time, that participant would have a higher level of physical activity than those who had never performed physical activities during work time. This finding provided evidence for the health benefits of promoting exercise behavior in the workplace. As earlier studies have shown enormous potential for health benefits when sedentary people can be persuaded to become moderately active [99,100], this result suggested that the office-based environment is an ideal setting for employee’s health intervention and well-being promotion [101]. As such, current results suggest that there is a great opportunity for facilitating exercise behavior to reduce sedentary behavior in the office context. In addition, our regression analysis showed that the underlying factors of Intrinsic Motivation, Social Environment, and Work Environment can positively influence office workers’ exercise behavior intention in office environments.

Our findings present several scientific implications. First, we proposed a measure and showed that it has quite strong internal validity. Thereby, the current research contributes an important measure to the scientific literature. We now have a sound basis for assessing determinants of office exercise behavior. At the same time, the current results confirm earlier research that investigates the determinants of exercise behavior in general (i.e., outside of the specific context of office exercise). That is, confirming earlier research [46,47,54,95], the current research shows that office exercise behavior is determined by enjoyment, competence, perceived health, break time, family and friends’ influence, colleague influence, and superior influence. We also explored how determinants relate to exercise intention using this measure. Because behavioral intention is deemed important within the literature on exercise behavior change [102,103,104], the factor structure found in this study can provide a useful model for future researchers seeking to develop predictive models concerning office workers’ exercise behavior and the facilitation of workplace exercise behavior. Future research investigating determinants of office exercise behavior can use our measure to study the relationship between these determinants and actual office exercise behavior. As the determinants of exercise differ by population subgroup [32], the current study increased the understanding of exercise determinants for office workers as a major population segment. The results of this study contribute to the domain of exercise behavior change by providing evidence on exercise determinants in the specific office-based context.

This study also has societal implications for promoting occupational health and workplace vitality. The determinants and underlying common factors of the OEBD scale found in this study provide a better understanding of exercise behavior in the office context. The measure we developed can be used in research on office exercise promotion programs. The strong relationship between common factors (Intrinsic Motivation, Social Environment, and Work Environment) and exercise behavior intention suggests that office exercise promotion programs need to consider these factors in their design. This study provides a basis for developing workplace health intervention, and moreover, contributes to solving the physical inactivity problem among office workers.

Our research had some limitations. However, the sampling method applied in this study allowed us to collect data from a wide range of participants. Thereafter, a measure of office exercise behavior determinants has been developed and demonstrated with promising reliability and validity. Yet international participants might have different cultural backgrounds and office norms and more research should be undertaken to fully understand the use and limitations of the OEBD Scale in various social and cultural situations. 

## 5. Conclusions

In conclusion, this study investigated the determinants of office workers’ exercise behavior and developed the Office Exercise Behavior Determinants Scale. The current research contributed to our understanding of existing exercise behavior determinants by providing a new measure of exercise behavior for the specific office context. In sum, these results allow measurement of determinants of physical exercise at work and thereby help improve the amount of exercise performed by office workers at the workplace and stimulate office worker’s physical and mental health.

## Figures and Tables

**Table 1:** Potential determinants of office exercise behavior.

	Potential Determinants	Concept Proposed by	Measure Developed by
1	Perceived Behavioral Control	Ajzen, 1991 [40]	Ajzen & Madden, 1986 [62]
2	Competence	Bandura, 1982 [42]	Buckworth, Lee, Regan, Schneider & DiClemente, 2007 [63]
3	Perceived Health	Maddux & Rogers, 1983 [49]	Sechrist, Walker & Pender, 1987 [64]
4	Enjoyment	Ryan, 1982 [50]	Buckworth, Lee, Regan, Schneider & DiClemente, 2007 [63]
5	Appearance	Lee, Nigg, Diclemente & Courneya, 2001 [51]	Buckworth, Lee, Regan, Schneider & DiClemente, 2007 [63]
6	Family and Friends Influence	Rogers, 1975 [52]	Taylor & Todd, 1995 [53]
7	Colleague Influence	Rogers, 1975 [52]	/
8	Superior Influence	Taylor & Todd, 1995 [53]	/
9	Social Support	Prochaska & DiClemente, 2005 [43]	Buckworth, Lee, Regan, Schneider & DiClemente, 2007 [63] Sechrist et al., 1987 [64]
10	Social Reinforcement	Dishman, Sallis & Orenstein, 1985 [54] Prochaska & DiClemente, 2005 [43]	Elliot et al., 2004 [65]
11	Vitality Tradition	Bartholomew et al., 2006 [55]	/
12	Public Space Scale	Tudor-Locke, Schuna, Frensham & Proenca, 2014 [56]	/
13	Exercise Facilities	Choi, Song, Edge, Fukumoto & Lee, 2016 [57]	/
14	Exercise Tutorial	Fogg, 2009 [58]	/
15	Work Pace	Bauman, Allman-Farinelli, Huxley & James, 2008 [59]	/
16	Break Time	Fogg, 2009 [58]	/
17	Policy of Working Company	Pronk & Kottke, 2009 [60]	/
18	Work Burden	Gorm & Shklovski, 2016 [61]	/

**Table 2:** The four-factor solution.

Factor	Initial Eigenvalue	Rotation Sums of Squared Loadings		N of Items	Cronbach’s Alpha
		**% of Variance**	**Cumulative %**		
1	9.0404	25.2850	25.2850	13	0.9435
2	4.1623	13.5438	38.8287	8	0.8491
3	3.2905	12.3931	51.2219	6	0.8746
4	2.2506	9.2420	60.4638	4	0.8403

**Table 3:** Factor loadings of items across factors.

Item Number	Description of Determinant	Factor 1 Intrinsic Motivation	Factor 2 Extrinsic Motivation	Factor 3 Social Environment	Factor 4 Work Environment
28	Enjoyment	0.8056	0.2635	0.0511	0.0574
14	Competence	0.8005	0.0472	0.1150	−0.0441
16	Competence	0.7954	0.0611	0.1505	−0.0600
29	Enjoyment	0.7843	0.2586	0.0746	0.1791
13	Competence	0.7767	0.1187	0.1745	−0.0629
33	Enjoyment	0.7687	0.2466	0.1516	0.1477
30	Enjoyment	0.7678	0.1300	−0.0081	0.0842
32	Enjoyment	0.7662	0.1434	−0.0768	0.1570
12	Competence	0.7616	0.1352	0.0376	−0.1002
34	Enjoyment	0.7418	0.3194	0.1099	0.1398
17	Competence	0.7078	0.1302	0.0705	−0.1487
15	Competence	0.7032	−0.0077	−0.0613	−0.0642
18	Competence	0.6952	−0.0295	−0.0685	−0.1779
23	Perceived Health	0.0801	0.7533	−0.0235	−0.0139
22	Perceived Health	0.1404	0.7391	−0.0213	0.0448
27	Perceived Health	0.1530	0.7174	0.0577	0.1294
20	Perceived Health	0.0300	0.6709	−0.0921	0.0076
25	Perceived Health	0.2201	0.6526	0.0549	0.0961
19	Perceived Health	0.3154	0.6500	−0.0327	−0.0577
24	Perceived Health	0.1730	0.6422	0.0574	−0.0600
21	Perceived Health	0.0219	0.6255	0.1580	0.1058
44	Colleague Influence	0.1325	−0.0097	0.8402	0.1179
46	Superior Influence	0.1236	−0.0249	0.8094	0.1354
45	Superior Influence	−0.0602	0.0841	0.8071	0.0063
43	Colleague Influence	−0.0755	0.1388	0.7954	0.0185
42	Family and Friend Influence	0.1519	0.0991	0.7142	0.1857
47	Superior Influence	0.1172	−0.1380	0.6615	0.0939
57	Work Pace	−0.0460	0.0551	0.0667	0.8071
59	Policy	0.0637	0.0047	0.2135	0.7996
60	Work Burden	−0.0841	0.1261	0.0727	0.7987
58	Break Time	0.0112	0.0154	0.1350	0.7926

**Table 4:** Results of confirmatory factor analysis.

	χ^2^	df	*p*	χ^2^/df	RMESA	CFI	SRMR
Model A	4938.9977	434	0.0000	11.3802	0.1474	0.4977	0.1531
Model B	3596.2163	433	0.0000	8.3053	0.1236	0.6473	0.1169
Model C	2944.3824	431	0.0000	6.8315	0.1105	0.7198	0.1039
Model D	2063.6010	428	0.0000	4.8215	0.0894	0.8176	0.0737

Note: RMESA = root mean square error of approximation. CFI = comparative fit index. SRMR = standardized root mean square residual.

**Table 5:** The composite reliability and average variance extracted of the four factors.

	AVE	CR
Intrinsic Motivation	0.5670	0.9439
Extrinsic Motivation	0.4220	0.8529
Social Environment	0.5432	0.8755
Work Environment	0.5711	0.8419

Note: CR = composite reliability. AVE = average variance extracted.

**Table 6:** The square root values of average variance extracted.

	Intrinsic Motivation	Extrinsic Motivation	Social Environment	Work Environment
Intrinsic Motivation	0.7530			
Extrinsic Motivation	0.371 **	0.6496		
Social Environment	0.156 **	0.085	0.7370	
Work Environment	0.017	0.112 *	0.263 **	0.7557

Note: * *p* < 0.05, ** *p* < 0.01.

## Data Availability

The data that support the findings of this study are available on request from the corresponding author, Xipei Ren. The data are not publicly available due to their containing information that could compromise the privacy of research participants.

## Appendix A. Questions Developed for the Online Survey Study

In this survey, you will be asked questions about physical exercise. Most people complete the survey in approximately 15 min. These details apply to your participation in this survey.

By participating in this survey, you agree to the terms of our consent form.

There are three parts to this questionnaire. Part 1 asks about your attitude about physical activity and exercise in your working environment. Part 2 asks about your current physical activities, your occupation and general demographics such as your age, nationality, and gender.

### Appendix A.1. Part 1: Potential Determinants and Items of office Exercise Behavior Determinants

**Perceived Behavioral Control**

1. I have control over whether I do or not do exercise.

2. For me to exercise is easy.

3. If I wanted to, I could easily exercise.

**Competence**

4. I think I am pretty good at physical activities.

5. I put a lot of effort into physical activity.

6. I think I do pretty well at physical activity, compared to my peers.

7. I haven’t tried very hard to do well at physical activities.

8. I try very hard at physical activity.

9. I am pretty skilled at the level of exercise that I do.

10. I haven’t put much energy into doing physical activity.

**Perceived Health**

11. Exercise improves my mental health.

12. Exercise increases my muscle strength.

13. Exercising will keep me from having high blood pressure.

14. My muscle tone is improved with exercise.

15. Exercising improves functioning of my cardiovascular system.

16. My disposition is improved with exercise.

17. Exercising helps me sleep better at night.

18. I will live longer if I exercise.

19. Exercising improves overall body functioning for me.

**Enjoyment**

20. I enjoy participating in exercise very much.

21. Exercise is fun to do.

22. I think that physical activity is boring.

23. I feel like I have to participate in physical activity.

24. Physical activity does not hold my attention at all.

25. I would describe physical activity as very interesting.

26. I think that physical activity is quite enjoyable.

27. While participating in physical activity, I think about how much I enjoy it.

**Appearance**

28. I exercise to keep my appearance.

29. I exercise to control my weight so that I look good for others.

30. People who are thin and successful probably have to exercise a lot.

31. I don’t want to look weak, so I try to work out a lot.

32. I exercise so that I will not look too fat or flabby.

**Family and Friends’ Influence**

33. My family and friends would think that I should do some physical activity.

34. Generally speaking, I want to do what my families and friends think I should do.

**Colleague Influence**

35. My colleagues would think that I should do some physical activity.

36. Generally speaking, I want to do what my colleagues think I should do.

**Superior Influence**

37. My superior would think that I should do some physical activity.

38. Generally speaking, I want to do what my superior think I should do.

39. I will have to do some physical activity because my superior requires it.

**Social Support**

40. People who are physically active are more popular than those who are not.

41. Exercising increases my acceptance by others.

**Social Reinforcement**

42. If I would have a partner who does physical exercise with me, it would be a reinforcement.

**Vitality Tradition**

43. The healthy tradition at my office influences my exercise behavior.

**Public Space Scale**

44. There will not be enough public activity space for exercise in my office.

45. For me, having enough public activity space for exercise in the office is important.

**Exercise Facilities**

46. There will not be enough exercise facilities for everyone in my office.

47. For me, having enough exercise facilities for everyone to use is important.

**Exercise Tutorial**

48. For me, having an exercise coach in the office is important.

**Work Pace**

49. The work pace in the office influences my exercise behavior.

**Break time**

50. The break time in the office influences my exercise behavior.

**Policy of Working Company**

51. The policy of company influences my exercise behavior.

**Work Burden**

52. The work burden in the office influences my exercise behavior.

**Behavior Intention**

53. I intend to do physical exercise.

54. I will try to do physical exercise in my office.

55. How regularly do you intend to do physical exercise in your office?

### Appendix A.2. Part 2: Physical Activity, Occupation and Demographics

1. During a typical 7-day period (a week), how many times on the average do you do the following kinds of exercise for more than 15 min during your free time (write on each line the appropriate number).

	**Times per Week**
**STRENUOUS EXERCISE** **(HEARTBEATS RAPIDLY)** (e.g., running, jogging, hockey, football, soccer, squash, basketball, cross country skiing, judo, roller skating, vigorous swimming, vigorous long-distance bicycling)	______
**MODERATE EXERCISE** **(NOT EXHAUSTING)** (e.g., fast walking, baseball, tennis, easy bicycling, volleyball, badminton, easy swimming, alpine skiing, popular and folk dancing)	______
**MILD EXERCISE** **(MINIMAL EFFORT)** (e.g., yoga, archery, fishing from riverbank, bowling, horseshoeing, golf without using a cart, snowmobiling, easy walking)	______

2. Have you ever performed physical activities during work time? (e.g., walking stairs; work out during lunch break; small physical activities)

□ Yes □ No

3. Your main occupation is _____

□ Paid full-time work

□ Paid part-time or casual work

□ Unemployed and looking for work

□ Studying or researching

□ Retired

□ Others

4. How would you classify your industry?

□ Agriculture, Forestry, Fishing and Hunting

□ Utilities

□ Construction

□ Manufacturing

□ Trade (Wholesale/retail trade)

□ Transportation and Warehousing

□ Finance and Insurance

□ Real Estate, Rental and Leasing

□ Information

□ Services

□ Professional, Scientific and Technical Services

□ Education

□ Health Care and Social Assistance

□ Arts, Entertainment, and Recreation

□ Government and Public Administration

□ Others

5. How would you classify your role?

□ Manager

□ Self-employed/Partner

□ Administrative Staff

□ Support Staff

□ Consultant

□ Trained Professional

□ Skilled Laborer

□ Researcher

□ Student

□ Temporary Employee

□ Others

6. The organization you work for is in which of the following:

□ Public sector (e.g., government)

□ Private sector (e.g., most businesses and individuals)

□ Non-profit sector

□ Don’t know

□ Others

7. What is your working environment?

□ Office

□ Home

□ Outdoor

□ Factory

□ Others

8. Please try to estimate: How many hours do you work per week?

□ Less than 10 h per week

□ 11–20 h per week

□ 21–30 h per week

□ 31–40 h per week

□ 41–50 h per week

□ 51–60 h per week

□ More than 60 h per week

9. Are you:

□ male

□ female

□ Non-binary

10. How old are you?

□ Under 20

□ 20–24

□ 25–29

□ 30–34

□ 35–39

□ 40–49

□ 50–59

□ 60 or over

11. How many years of formal school education (or their equivalent) did you complete (starting with primary school)?

□ 10 years or less

□ 11 years

□ 12 years

□ 13 years

□ 14 years

□ 15 years

□ 16 years

□ 17 years

□ 18 years or over

## Appendix B. The Office Exercise Behavior Determinants

**Table A1:** The finalized 32-item Office Exercise Behavior Determinants Scale.

**Factors**	**Determinants**	**Items**
Intrinsic Motivation	Competence	1. I think I am pretty good at physical activities. 2. I put a lot of effort into physical activity. 3. I think I do pretty well at physical activity, compared to my peers. 4. I haven’t tried very hard to do well at physical activities. 5. I try very hard at physical activity. 6. I am pretty skilled at the level of exercise that I do. 7. I haven’t put much energy into doing physical activity.
	Enjoyment	8. I enjoy participating in exercise very much. 9. Exercise is fun to do. 10. I think that physical activity is boring. 11. Physical activity does not hold my attention at all. 12. I would describe physical activity as very interesting. 13. I think that physical activity is quite enjoyable. 14. While participating in physical activity, I think about how much I enjoy it.
Extrinsic Motivation	Perceived Health	15. Exercise improves my mental health.16. Exercise increases my muscle strength. 17. Exercising will keep me from having high blood pressure. 18. My muscle tone is improved with exercise. 19. Exercising improves functioning of my cardiovascular system. 20. My disposition is improved with exercise. 21. Exercising helps me sleep better at night. 22. Exercising improves overall body functioning for me.
Social Environment	Family and Friends’ Influence	23. Generally speaking, I want to do what my families and friends think I should do.
	Colleague Influence	24. My colleagues would think that I should do some physical activity. 25. Generally speaking, I want to do what my colleagues think I should do.
	Superior Influence	26. My superior would think that I should do some physical activity. 27. Generally speaking, I want to do what my superior think I should do. 28. I will have to do some physical activity because my superior requires it.
Work Environment	Work Pace	29. The work pace in the office influences my exercise behavior.
	Break time	30. The break time in the office influences my exercise behavior.
	Policy of Working Company	31. The policy of company influences my exercise behavior.
	Work Burden	32. The work burden in the office influences my exercise behavior.
